# Depressive symptoms in Chinese family caregivers of patients with heart failure: A cross-sectional study

**DOI:** 10.1097/MD.0000000000006480

**Published:** 2017-03-31

**Authors:** Xiaolin Hu, Wenxia Huang, Yonglin Su, Moying Qu, Xingchen Peng

**Affiliations:** a Department of Nursing; b Department of Healthcare; c Department of Rehabilitation; d Department of Cardiology; e Department of Medical Oncology, Cancer Center, State Key Laboratory of Biotherapy, West China Hospital, Sichuan University, Chengdu, Sichuan, P. R. China.

**Keywords:** depressive symptoms, factors, family caregivers, heart failure

## Abstract

Depressive symptoms are related to negative health outcomes in caregivers of patients with HF. Understanding the factors that are associated with depressive symptoms among caregivers is essential to providing appropriate interventions. Little is known about which status and factors are related to depressive symptoms among Chinese caregivers of patients with heart failure. This study aimed to investigate the status of depressive symptoms and to identify the factors that are associated with depressive symptoms in family caregivers of patients with heart failure in China.

A cross-sectional design and a convenience sample were used. Participants (N = 134) from 1 hospital in Chengdu were recruited from June 2013 to June 2014. The following measurement tools were used in this study: Center for Epidemiologic Studies Depression Scale, Hospital Anxiety and Depression Scale, Coping Strategies Simplified Coping Style Questionnaire, and Zarit Burden Interview. A hierarchical multiple linear regression analysis was used to determine which factors were associated with depressive symptoms.

The results showed that 31% of the caregivers experienced depressive symptoms. The type of payment for treatment (*b* = −0.312, *P* < 0.01), readmissions within the last 3 months (*b* = 0.397, *P* < 0.01), duration of caregiving (*b* = −0.213, *P* < 0.05), caregiver burden (*b* = 0.299, *P* < 0.05), active coping (*b* = −0.235, *P* < 0.01), and negative coping (*b* = 0.245, *P* < 0.05) were related to caregivers’ depressive symptoms. Fifty-four percent of the variance in caregivers’ depressive symptoms was explained by these factors.

The caregiver depressive symptoms in China were higher than those reported in studies that were conducted in Western countries. Caregiver depressive symptoms can be improved by providing support for new caregivers (with a caregiving duration of less than 1 year), reducing readmissions, easing caregiver burden, and promoting their coping strategies.

## 1 Introduction

Family caregivers play a crucial role in the delivery of care to patients with heart failure (HF). However, caregivers of patients with HF have poor mental health and a higher level of emotional distress.^[1]^ Depression is a common health outcome (along with emotional distress and negative psychological consequences) in the caregiver literature.^[2,3]^ The prevalence of caregiver depressive symptoms in association with HF (21%) was at least comparable with the results of a meta-analysis of 57 other caregiver studies.^[4]^ The depressive symptoms among caregivers were equal to or even worse than those of patients with HF.^[5,6]^ In addition, the primary caregivers of HF patients who reported depressive symptoms at baseline had poor mental quality of life at 8-month follow-up.^[7]^ Moreover, caregivers’ depressive symptoms have negative effects not only on their own health but also on patients’ quality of life.^[1,6]^

Literature on the caregiver population has shown that many factors affect caregiver depressive symptoms, including patient characteristics,^[8,9]^ caregiver characteristics,^[10,11]^ objective caregiving load,^[12]^ caregiver burden,^[9,11]^ perceived control,^[9,11]^ and coping strategies.^[13,14]^

Despite the increasing prevalence of HF and diminished mental health of caregivers, to date, most of the caregiver literature has focused on other burdensome diseases such as stroke and cancer, rather than HF. There is a relative disregard in the field of caregivers of patients with HF. The available literature from Western countries has demonstrated that caregivers of patients with HF have a higher level of depressive symptoms.^[15,9]^ Caregivers’ functional status, burden, and perceived control were associated with depressive symptoms among caregivers of patients with HF.^[9]^ In China, due to a lack of formal institutions for long-term care and Chinese cultural norms of Confucianism (e.g., filial obligations and interdependence in family members), most patients with chronic diseases receive home-based care from family caregivers. Thus, Chinese family caregivers have to have greater responsibilities and experience more burden in association with caregiving, which increases their vulnerability to depression. So far, however, few studies have investigated the status and factors that are associated with depressive symptoms among Chinese caregivers of patients with HF. Given the emotional distress among caregivers and the relationship to the wellbeing of patients, deep investigation of the factors that are associated with caregiver depressive symptoms is warranted to provide appropriate support for Chinese caregivers of patients with HF to improve their own and patients’ wellbeing.

Therefore, the specific objectives of this study were to investigate the status of depressive symptoms among caregivers of patients with HF in China and identify the factors that are associated with depressive symptoms.

## 2 Methods

### 2.1 Study design

A cross-sectional descriptive design and convenience sampling were used from June 2013 to June 2014 at the cardiac inpatient ward of 1 regional teaching hospital in Chengdu, China. This study was approved by the Medical Ethics Committees of Sichuan University. Written informed consent was obtained from all the participants.

### 2.2 Sample

The inclusion criteria for the patients were: 18 years of age or older and having a primary diagnosis of HF. One primary family caregiver for each patient was identified to participate in the study. The inclusion criteria for the caregivers were: over 18 years of age, being the primary family caregiver with the longest duration of the provision of care to the patient, and being able to speak Chinese. Paid caregivers and caregivers with cognitive impairment were excluded.

### 2.3 Measures

Participants’ data were collected at the inpatient cardiac ward in the hospital prior to discharge, including participants’ characteristics, depressive symptoms, objective caregiving burden, subjective caregiving burden, and caregivers’ coping ability. The cardiac nurses on our team identified potentially eligible participants and explained the study to them in an in-person interview. Written consent forms were signed by the enrolled participants. Clinical data were obtained from medical records. The majority of the participants (90.3%) completed the surveys via a self-report questionnaire. For the 9.7% of participants who were illiterate, a researcher read the survey questions to them and then recorded their answers.

The instruments that were used in this study included: a demographic survey, the Center for Epidemiologic Studies Depression Scale (CES-D), the coping strategies simplified coping style questionnaire (SCSQ), and the zarit burden interview (ZBI).

### 2.4 Demographic survey

#### 2.4.1 Characteristics of the patients

Data were collected on the following patient characteristics: sex, age, education level, marital status, type of payment for treatment, New York Heart Association (NYHA) classification, duration of HF, and readmissions within the last 3 months.

#### 2.4.2 Characteristics of the caregivers

Data were collected on the following family caregiver characteristics: sex, age, education level, marital status, relationship to the patient, coresidency with the patient, employment, monthly family income, and chronic diseases.

#### 2.4.3 Caregivers’ depressive symptoms

Caregivers’ depressive symptoms were evaluated using the CES-D scale.^[16]^ The CES-D has 20 items and has a 4-point scale ranging from 0 (“rarely”) to 3 (“most of the time”). The total score ranges from 0 to 60, with a higher score indicating a higher level of depressive symptoms. The Chinese version of the CES-D has adequate reliability and validity among Chinese populations.^[17,18]^ A score of 16 or above is an indicative of probable depressive symptoms.^[16,19]^

#### 2.4.4 Coping ability

Caregivers’ coping ability was measured using the 20-item SCSQ.^[20,21]^ The SCSQ was developed to measure the coping style of Chinese people. It has 2 subscales, including the 12-item active coping strategies subscale and the 8-item negative coping strategies subscale. The 4-point scale ranges from 0 (“rarely”) to 3 (“always”). A higher score on the active coping strategies subscale is indicative of better coping ability, whereas a higher score on the negative coping strategies subscale is indicative of poorer coping ability. Cronbach alpha coefficients for the active and negative coping strategies subscales were 0.89 and 0.78, respectively.

#### 2.4.5 Subjective caregiving burden

Subjective caregiving burden was measured using the 22-item ZBI.^[22]^ The ZBI has a 5-point scale ranging from 0 (“never”) to 4 (“almost always”). The total score ranges from 0 to 88, with a higher score indicating a heavier burden. The Chinese version of the ZBI has adequate reliability and validity.^[23]^

#### 2.4.6 Objective caregiving burden

Objective caregiving burden in this study was examined using 3 indexes: the duration of caregiving, number of caregiving hours per day, and number of caregivers.

### 2.5 Data analysis

SPSS 16.0 (SPSS Inc, Chicago, IL) was used to perform the data analyses. Descriptive statistics were used to describe the participants’ demographic and clinical features. Correlation analyses were used to examine the relationships among depressive symptoms and readmissions, caregiver burden, and coping ability. A hierarchical multiple linear regression model was used to test the relationships between the factors and depressive symptoms and examine the proportion of variance in depressive symptoms explained by the independent variables. The CES-D scores were the dependent variables in the analyses. The independent variables, including patient characteristics, caregiver characteristics, objective caregiving burden, subjective caregiving burden, and coping ability, were entered into the model in the following order: Step 1: characteristics of the patients; Step 2: characteristics of the caregivers; Step 3: objective caregiving burden; Step 4: subjective caregiving burden; and Step 5: coping ability. The analyses were performed in stages by including these 5 blocks successively. The contribution of each block to explaining the variance in caregivers’ depressive symptoms was shown by the change in *R*^2^. All the tests were 2-tailed, and a *P* value less than 0.05 was considered to be statistically significant.

## 3 Results

Of the 150 potentially eligible participants whom we contacted, 6 were not willing to participate in the study, 5 declined to participate due to a tight schedule, and 5 declined to participate without providing a reason. A total of 134 participants who completed the surveys were recruited for the study.

### 3.1 Sample characteristics

#### 3.1.1 Patients

As shown in Table 1, the average age of the patients was 66.3 years (SD *=* 15.2). Most of them (71.6%) were older than 60 years of age. The majority of them were men (64.2%) and married (91.0%). Most of them (55.2%) had an education level of primary school or below. The majority of them (80.6%) had medical and other types of insurance. NYHA Classifications of the patients were: Class I: 5 patients (3.7%); Class II: 11 patients (8.2%); Class III: 81 patients (60.5%); and Class IV: 37 patients (27.6%). Most of them (73.1%) had been afflicted with HF for more than 1 year. The average number of readmissions within the last 3 months was 1.47 (SD *=* 0.77).

**Table 1:**
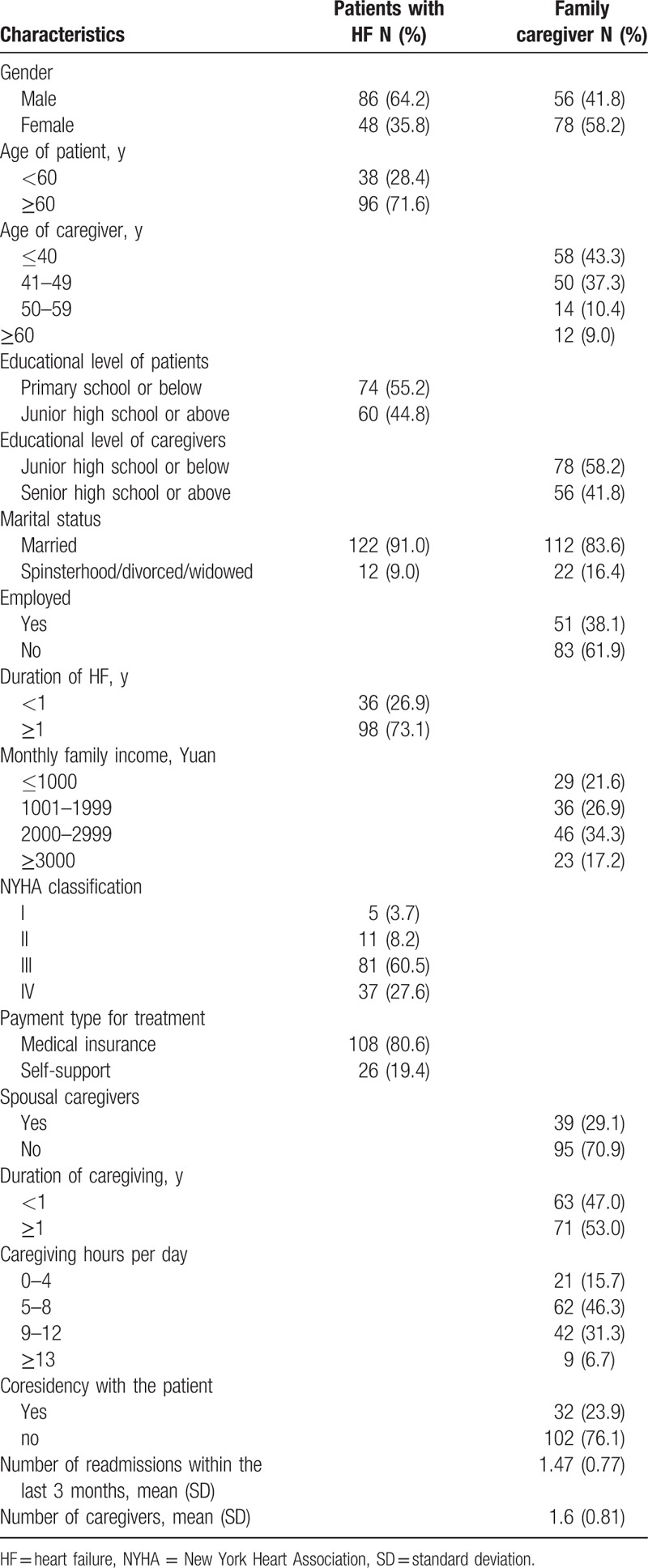
Characteristics of patients and their family caregivers (N = 134).

#### 3.1.2 Caregivers

The average age of the primary family caregivers was 41.4 years (SD = 13.6). The majority of them (80.6%) were younger than 50 years of age. Most of them were female (58.2%) and married (83.6%). The majority of them (58.2%) had an education level equivalent to junior high school or below. Most of them (82.8%) had a monthly family income of less than 500 US dollars. Spousal caregivers accounted for 29.1% of the sample. Most of the caregivers were unemployed (61.9%) and living with the patient (23.9%). The majority of them (84.3%) spent more than 4 hours per day caregiving. Most of them (53.0%) provided care to the patient for more than 1 year. The average number of caregivers was 1.6 (SD = 0.81).

### 3.2 Caregiver depressive symptoms

The average CESD score was 13.06 (SD = 3.18) in this study. The majority of the caregivers (68.7%) had a CESD score of less than 16, whereas 31.3% of the caregivers had a CESD score of more than 16, indicating that they were suffering from depressive symptoms (Table 2).

**Table 2:**
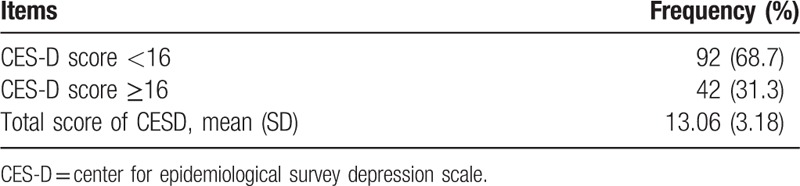
Depressive symptoms among family primary caregivers (N = 134).

### 3.3 Relationships among depressive symptoms and readmissions, caregiving burden, active coping, and negative coping

The Pearson correlation analyses demonstrated that depressive symptoms were positively associated with readmissions (*r* = 0.497, *P* < 0.01), caregiver burden (*r* = 0.278, *P* < 0.01), and negative coping (*r* = 0.403, *P* < 0.01) and inversely associated with active coping (*r* = −0.346, *P* < 0.01) (Table 3).

**Table 3:**
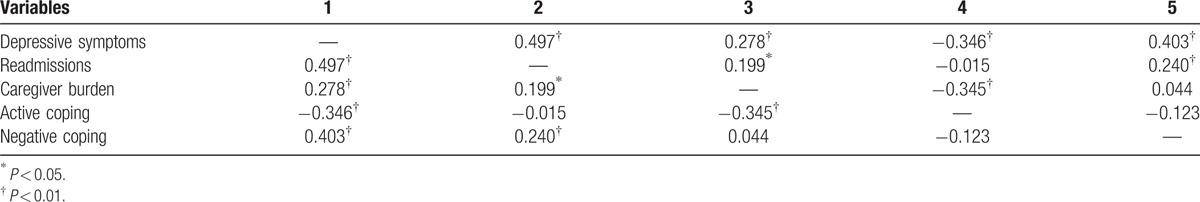
Relationship between depressive symptoms, readmission, caregiver burden, and coping (N = 134).

### 3.4 Hierarchical multiple regression analyses with depressive symptoms

The hierarchical multiple regression (HMR) models were used to determine which factors were associated with depressive symptoms among primary family caregivers. The CESD scores served as the dependent variables. The 5 blocks of independent variables, including patient characteristics, caregiver characteristics, objective caregiving burden, subjective caregiving burden, and coping ability, were entered into the model successively as independent variables. As shown in Table 4, except for caregivers’ characteristics (the *P* values for all caregivers’ variables were greater than 0.05), the other blocks of independent variables made a significant contribution to explaining the variance in caregivers’ depressive symptoms. The incremental change in *R*^2^, the proportion of variance explained by each block of variables, was 29.0%, 2.0%, 3.0%, 9.5%, and 10.5% for patient characteristics, caregiver characteristics, objective caregiving burden, subjective caregiving burden, and coping ability, respectively. The type of payment for treatment (*b* = −0.312, *P* < 0.01), readmissions within the last 3 months (*b* = 0.397, *P* < 0.01), duration of caregiving (*b* = −0.213, *P* < 0.05), caregiver burden (*b* = 0.299, *P* < 0.01), active coping (*b* = −0.235, *P* < 0.01), and negative coping (*b* = 0.245, *P* < 0.01) were significantly related to caregivers’ depressive symptoms. Fifty-four percent of the variance in caregivers’ depressive symptoms was explained by these factors.

**Table 4:**
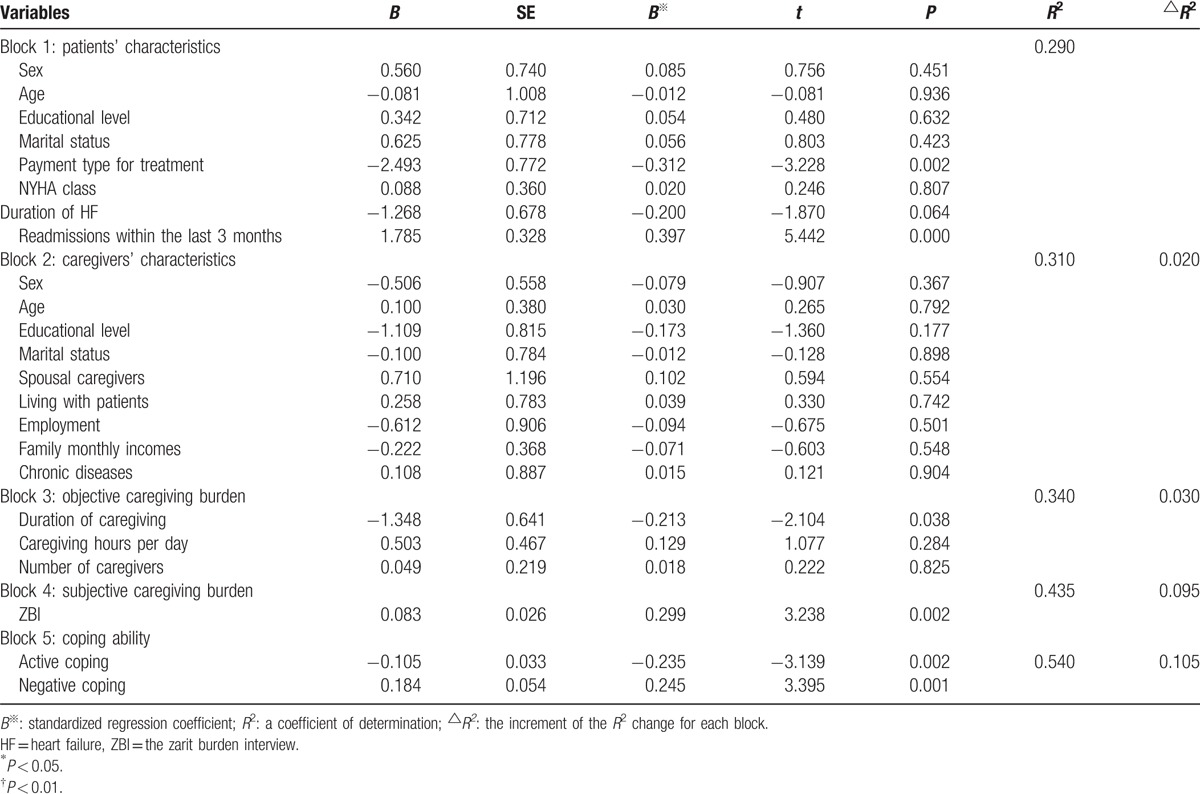
Factors related to depressive symptoms in the Hierarchical Multivariate Regression analysis (N = 134).

## 4 Discussion

This study revealed that more than one-quarter of the caregivers (31%) of patients with HF in our study experienced depressive symptoms, which is higher than the proportions presented in other studies that were conducted in Western countries. The factors that were associated with depressive symptoms included type of payment for treatment, readmissions within the last 3 months, duration of caregiving, caregiver burden, and coping ability.

The mean CES-D score in our sample was 11.4 (SD = 8.8), and more than one-quarter of the caregivers (31%) of patients with HF experienced depressive symptoms. The CES-D score and prevalence were higher than those who were presented in previous studies in this field from Western countries.^[9,15]^ Pinquart and Sörensen^[24]^ demonstrated that caregivers in Asian countries were more distressed by depressive symptoms than Caucasians. Our results were consistent with those of Pinquart and Sörensen.^[24]^ Culture has a great impact on individuals’ caregiving experiences, perceptions, and behaviors. The higher depressive symptoms observed in the present study may have been related to Chinese cultural norms, which are influenced by Confucianism. Confucianism emphasizes the ideas of family first and filial piety. In this context, the provision of care to patients is an obligation of family members, even if they are at risk of diminished psychological and physical health. A strong emphasis on authority and familism instead of reciprocal affective ties in caregiving may increase the level of emotional distress for family caregivers.^[24,25]^ In addition, Chinese caregivers tend to be more conservative with regard to expressing their true feelings about caregiving, especially when they are negative such as “burnout.”^[23]^ Most of them sacrifice themselves for their families. The complicated conflicts relating to the obligation and suppressed expression of emotion make them more vulnerable to depression. These findings reveal a specific aspect of caregiving that might be the focus of future research on Chinese people.

Patient characteristics, including readmissions and financial types for treatment, made the most important contributions to the interpretation of depressive symptoms, accounting for 28.5% of the variance. The type of payment for treatment was related to depressive symptoms. Caregivers who provided care to patients with medical and other types of insurance experienced lower depressive symptoms than those who were self-paying. Lower financial status was linked to increased caregiver burden.^[26,27]^ Financial burden was one of the important factors that was associated with caregivers’ perception of depression, especially for low-income families. In our study, most of the caregivers had a low financial status and, therefore, had difficulties in covering the high medical expenses without reimbursement from medical and other insurance. As a result, strategies that are intended to increase the coverage and ratio of reimbursement of medical insurances are needed to improve depressive symptoms.

Readmissions were associated with depressive symptoms among caregivers of patients with HF in this study. Caregivers who provided care to patients with frequent readmissions were more vulnerable to depression. The results were consistent with those of Schwarz and Elman,^[15]^ who reported that an increased risk of readmission was related to the interaction between caregiver depression and stress. Frequent readmissions exerted a heavy burden on family caregivers due to their association with aggravating symptoms, higher caregiving requirements, and increased economic burden.^[28]^ Depressive symptoms occur when caregivers have unmet needs and multiple stresses caused by frequent readmissions, which, in turn, have a negative impact on their ability to provide care to the patient. Insufficient information and self-care skills among patients and their caregivers are related to readmissions.^[29–31]^ Thus, a detailed discharge plan that integrates knowledge and skills for patients and their caregivers may be an effective method to reduce readmissions as a means of improving caregivers’ depressive symptoms.

In the block with objective caregiving burden, only the duration of caregiving was associated with caregiver depressive symptoms. Caregivers who took care of patients for less than 1 year had a higher level of depressive symptoms than those with a longer duration of caregiving. Studies in this field have been inconsistent. Normally, an increased duration of caregiving was related to greater stress and emotional problems due to higher caregiving requirements and physician exhaustion. Shua-Haim et al^[12]^ reported that caregivers’ depressive symptoms increased over the caregiving trajectory, whereas McConaghy and Caltabiano^[32]^ found that the duration of caregiving was positively correlated with perceived wellbeing. The findings were similar to ours. Caregiver burden and distress were related to caregiving tasks as well as the perceived difficulty of performing caregiving. Caregivers who took care of patients for less than 1 year may have perceived more severe burden due to the lack of knowledge and skills with respect to caregiving,^[33]^ which may have increased their risk of developing depressive symptoms. Additionally, a lower duration of caregiving might make it difficult for caregivers to adapt to the caregiver role, increasing their emotional distress. Therefore, more attention and support are needed for new caregivers who have taken care of patients for less than 1 year.

In the current study, the caregivers’ subjective caregiving burden made a greater contribution to explaining the variance in depressive symptoms (9.5%) compared with objective caregiving burden (3.0%). The findings indicated that caregivers’ depressive symptoms were determined to a greater extent by their perceived caregiving burden than their objective burden, which is similar to the findings of previous studies.^[11,34]^ Caregivers’ depressive symptoms were associated with their appraisal of the caregiver role^[35]^ and their perceived difficulty in performing caregiving.^[34]^ Caregivers’ appraisal of caregiving differs from person to person, even when facing the same caregiving task.^[9]^ Caregivers who appraise caregiving activities and their ability negatively tend to be more vulnerable to depression. Insufficient support or coping strategies are contributors to caregivers’ negative appraisal of caregiving activities. Therefore, strategies that are intended to strengthen support and coping ability among caregivers are beneficial to the improvement of depressive symptoms.

We observed that active coping was negatively associated with depressive symptoms, whereas negative coping was positively related to caregivers’ depressive symptoms. Coping is presumed to mediate the relationship between emotional distress and stressful situations.^[36]^ Active coping strategies focus on problem solving to help to reduce distress and depressive symptoms.^[37,38]^ Caregivers who adopted more active coping strategies had better wellbeing and reported less stress and depressive symptoms.^[39,40]^ In contrast, passive coping strategies based on emotional coping instead of problem solving might lead to an accumulation of negative emotion, then aggravate emotional distress. Caregivers who used negative coping strategies frequently had reduced mental and physical health.^[41,42]^ Thus, strategies that are intended to improve their coping ability are necessary.

## 5 Limitations

This study was limited by the use of a cross-sectional design, which did not allow us to determine the causal relationships between the independent variables and depressive symptoms. The recruitment of a convenience sample from 1 hospital was another limitation that impacted the generalizability of the results to other caregiver populations. A multicenter sample and longitudinal study are recommended in future studies. Moreover, patients’ activities of daily living, caregivers’ perceived control, and social support should be taken into consideration, as they are factors that potentially affect depressive symptoms.

## 6 Implications

Early assessment and psychological intervention for caregivers of patients with HF should be completed to identify and help caregivers who are at high risk of depressive symptoms. In addition, the government should play a more important role in the provision of support to family caregivers through sponsored policies and strategies such as increasing the coverage and ratio of medical insurance. Moreover, the impact of culture on caregiver psychological wellbeing should be taken into consideration in the intervention to provide appropriate support for Chinese caregivers to optimize efficacy. Proposed strategies include encouraging caregivers to express and share their feelings about caregiving, providing necessary coping skills training, and helping them to understand the caregiver role in an appropriate way.

## 7 Conclusion

This study examined a vulnerable caregiver population with a high risk of depressive symptoms. The type of payment for treatment, patient readmissions, duration of caregiving, caregiver burden, and coping strategies were associated with caregiver depressive symptoms. These findings indicate that the caregivers of patients with HF who experience depressive symptoms may benefit from interventions that increase the coverage and ratio of medical insurance, reduce readmissions, address caregiver burden, and improve coping strategies. More attention should be paid to new family caregivers who have been providing care to a patient for less than 1 year.
